# Brown adipose tissue facilitates the fever response following infection with *Salmonella enterica* serovar Typhimurium in mice

**DOI:** 10.1016/j.jlr.2024.100617

**Published:** 2024-08-09

**Authors:** Mohan Li, Marina Barros-Pinkelnig, Günter Weiss, Patrick C.N. Rensen, Sander Kooijman

**Affiliations:** 1 Division of Endocrinology, and Einthoven Laboratory of Experimental Vascular Medicine, Deparment of Medicine, Leiden University Medical Center, Leiden, The Netherlands; 2 Department of Internal Medicine II, Medical University of Innsbruck, Innsbruck, Austria

**Keywords:** brown fat, nonshivering thermogenesis, fever, lipoprotein metabolism, bacterial infection

## Abstract

Brown adipose tissue (BAT) combusts lipids and glucose to generate heat. Via this process of nonshivering thermogenesis, BAT plays a pivotal role in thermoregulation in cold environments, but its contribution to immune-induced fever is less clear. Male APOE∗3-Leiden.CETP mice, a well-established model for human-like lipoprotein metabolism, and wild-type mice were given an intraperitoneal injection of *Salmonella enterica serovar* Typhimurium (S.tm). Energy expenditure and substrate utilization, plasma lipid levels, fatty acid (FA) uptake by adipose tissues, and lipid content and thermogenic markers in adipose tissues were examined. S.tm infection led to a set of characteristic symptoms, including elevated body temperature and decreased body weight. Whole-body energy expenditure was significantly decreased 72 h postinfection, but fat oxidation was increased and accompanied by a substantial reduction in plasma triglyceride (TG) levels as demonstrated in APOE∗3-Leiden.CETP mice. S.tm infection strongly increased uptake of FAs from TG-rich lipoproteins by BAT, which showed a positive correlation with body temperature in infected mice. Upon histological examination of BAT from wild-type or APOE∗3-Leiden.CETP mice, elevated levels of tyrosine hydroxylase were observed, indicative of stimulated sympathetic activity. In addition, the gene expression profile was consistent with more adrenergic stimulation, while lipid content was reduced. Furthermore, browning of white adipose tissue was observed, evidenced by a modest increase in TG-derived FA uptake, the presence of multilocular cells, and induction of uncoupling protein 1 expression. We proposed that BAT, or thermogenic adipose tissue in general, is involved in the maintenance of elevated body temperature upon invasive bacterial infection.

A growing body of research shows that adipose tissue has functions that go beyond energy storage; it serves as an endocrine organ, regulates inflammatory responses, and is involved in thermoregulation. For the latter, we distinguish between the insulating properties of white adipose tissue (WAT) and the heat production by brown adipose tissue (BAT). While white adipocytes store triglycerides (TGs) in large lipid droplets, brown adipocytes use TG for heat production facilitated by a high density of mitochondria that express uncoupling protein 1 (UCP-1). BAT is also densely innervated by the sympathetic nervous system (SNS), likely to facilitate a rapid response to environmental cold. Interestingly, WAT acquires features of BAT in response to prolonged cold exposure, a process known as "browning" (1).

Fever is an umbrella indicator of inflammation and infectious diseases. It is the outcome of coordinated autonomic reactions, including peripheral vasoconstriction, reduced sweating, shivering, and nonshivering thermogenic processes (2). The fact that the inflammatory cytokines interleukin-1β (IL-1β) and tumor necrosis factor α enhance whole-body oxygen consumption supports the idea that immune-induced fever actively increases thermogenesis (3). The question remains, however, to what extent BAT is involved in this fever response. Chemokine ligand 22 has been reported as a prostaglandin-dependent pyrogen, acting on the hypothalamus to stimulate sympathetic outflow to BAT and to induce hyperthermia (4). These data are in line with earlier reports indicating a crucial role for the SNS in endotoxin- and IL-1β-induced fever causation (5, 6). However, experiments in UCP-1 knockout mice suggested little to no involvement of BAT in pyrogenic effects (7, 8).

In the present study, we used infection with the gram-negative bacterium, *Salmonella enterica* serovar Typhimurium (S.tm) to study the involvement of BAT in infection-induced fever and employed APOE∗3-Leiden.CETP transgenic mice as a well-established model of human-like lipoprotein metabolism (9) to investigate the consequences of bacteremia for lipid metabolism. Our data strongly suggest that infection promotes thermogenic activity in BAT via the SNS, for which it takes up large quantities of TG-derived fatty acids (FAs) from circulating TG-rich lipoproteins (TRLs), resulting in strongly reduced blood TG levels.

## Materials and Methods

### Mice

All mouse experiments were reviewed and approved by Animal Welfare Body Leiden (IvD Leiden) and executed under a license (i.e., AVD11600202010187) granted by the Central Authority for Scientific Procedures on Animals in accordance with the Dutch Act on Animal Experimentation and EU Directive 2010/63/EU. The experiments were executed at Leiden University Medical Center. In both experiment, mice were housed under standard conditions, namely at a room temperature of 22°C, in a 12/12-h light/dark cycle, and with ad libitum access to food and water, unless indicated otherwise.

For experiment 1, male APOE∗3-Leiden.CETP mice (C57BL/6J background) were generated as previously described (9). At the age of 8–12 weeks, regular chow diet was replaced with a Western-type diet containing 0.25% cholesterol and 16% fat (Altromin, Lage, Germany). After 3 weeks on Western-type diet, body weight was determined, and 4-h fasted blood was obtained from the tail vein. The mice were then divided into two groups that were balanced for body weight and plasma TG and total cholesterol (TC) levels (see below) by using RandoMice software version 1.0.9 (10). On the day prior to the infection, all mice were individually housed.

In experiment 2, male C57BL/6J mice (Charles River, Saint-Germain-Nuelles, France) were used. At 10 weeks of age, body weight was determined, and 4-h fasted blood was obtained from the tail vein. The mice were divided into two groups balanced for body weight and plasma TG levels. On the day prior to the infection, all mice were individually housed.

### *In vivo* infection

S.tm (ATCC 14028) was cultured in Luria-Bertani medium in sterile conditions until reaching an *A*_600_ of 0.5–0.6, when the bacteria were in a logarithmic growth phase. Mice assigned to the infected group received a single intraperitoneal injection with 500 colony-forming units of S.tm in 200 μl of saline as described (11). Mice assigned to the control group received 200 μl of saline.

### Indirect calorimetry

In experiment 1, immediately after the intraperitoneal injection with S.tm or saline, the home cages with the individually housed mice were transferred to the indirect calorimetric system (Promethion line, Sable Systems International, Las Vegas, NV). In this system, O_2_ consumption, CO_2_ production, and physical activity (based on beam breaks) were recorded in 5-min bins. From these data, energy expenditure, fat oxidation, and carbohydrate oxidation rates were calculated (12).

### Plasma lipids measurement

Plasma TG and TC were measured using Cobas Triglycerides (106571) or Cobas Total Cholesterol (106570) enzymatic kits (Roche Diagnostics, Mannheim, Germany). Sample (7.5 μl) was mixed with 200 μl reagent (undiluted for TG and 3× diluted in PBS for TC) and incubated at room temperature for 30 min. Absorption was measured at 492 nm versus 650 nm for TG or at 505 nm versus 650 nm for TC.

### TG-derived FA, lipoprotein remnant, and glucose uptake by organs

Three days postinfection, mice received an intravenous injection with an emulsion of TRL-like particles (80 nm; 1 mg TG in 200 μl saline per mouse), prepared as described previously (13). In experiment 1, TRL-like particles were double-labeled with glycerol tri[^3^H]oleate ([^3^H]TO) and [^14^C]cholesteryl oleate ([^14^C]CO) to evaluate tissue uptake of TG-derived FAs and the tissue uptake of the particle core remnant, respectively. In experiment 2, 2-[^14^C]deoxyglucose was added to the emulsion of TRL-like particles labeled with [^3^H]TO. Mice were euthanized 15 min after the injection and perfused for 5 min with ice-cold PBS, after which tissues were harvested. Smaller tissues were dissolved in 0.5 ml while larger tissues were dissolved in 1 ml solvable (PerkinElmer) overnight at 56°C. Five milliliter of Ultima Gold scintillation liquid (PerkinElmer) was added to assess ^3^H- and ^14^C-activity using a Tri-Carb 2910TR Low Activity Liquid Scintillation Analyzer (PerkinElmer). Data were expressed as percentage of the injected dose per gram tissue.

### Histological examination of adipose tissue

Interscapular brown adipose tissue (iBAT) and subscapular brown adipose tissue (sBAT) were dissected from the dorsal and ventral of the scapula, respectively. Care was taken to separate BAT from surrounding WAT in case of iBAT or muscle in case of sBAT. Subcutaneous white adipose tissue (sWAT) was collected from the inguinal area, and embedded lymph nodes were removed.

For histological assessment, 5 μm tissue sections of sBAT and sWAT were stained with hematoxylin and eosin by using conventional procedures. Additional sections were dewaxed, rehydrated, and treated with peroxidase in order to stain for UCP-1 or tyrosine hydroxylase (TH). Antigen retrieval was accomplished in citrate buffer (10 mmol/L, pH 6.0). Slides were treated with normal goat serum (UCP-1) or BSA (TH) and incubated at 4°C overnight with an anti-UCP-1 antibody (1:4,000; Ab10983; Abcam, Cambridge, UK) or an anti-TH antibody (1:2,000; Ab112; Abcam). Afterward, sections were incubated for 30 min with a biotinylated goat α-rabbit secondary antibody (UCP-1; 1:600; Vector Labs, Burlingame, CA) or a DAKO EnVision anti-rabbit antibody (DAKO, Glostrup, Denmark). Using the Vector Laboratories Elite ABC kit (Vector Labs), the immunostaining was amplified, and Nova Red (Vector Labs) was used to visualize the immunoperoxidase complex. As a counterstain, Mayer's hematoxylin (1:4) was used.

### Gene expression analysis

RNA was extracted from frozen samples by lysing and homogenizing them using TriPure RNA Isolation Reagent (11667165001, Sigma Aldrich, Saint Louis, MO) and a FastPrep-24™ 5G bead beating grinder and lysis system (4.0 m/s for 10 s; MP Biomedicals™, Santa Ana). Subsequently, cDNA was synthesized from 1 μg of RNA using M-MLV Reverse Transcriptase (M1705, Promega, Madison, WI). Quantitative PCR was performed using a SYBR Green kit (Promega, Madison, WI) and a CFX96 PCR machine (Bio-Rad, Hercules, CA) following the manufacturers’ instructions. Gene expression levels were normalized to *β actin* and presented relative to the control mice.

### Protein quantification

Frozen samples were lysed with RIPA buffer (150 mM sodium chloride, 1.0% Triton X-100, 0.5% sodium deoxycholate, 0.1% sodium dodecyl sulfate, and 50 mM Tris pH 8.0) containing protease and phosphatase inhibitors (A32959, Thermo Fisher Scientific, Waltham, MA) and then homogenized using a FastPrep-24™ 5G bead beating grinder and lysis system (4.0 m/s for 10 s; MP Biomedicals™, Santa Ana, CA). The lysates were centrifuged (16.2 g for 5 min at 4°C) multiple times to remove lipids. Protein concentrations were determined using the Pierce™ BCA Protein Assay Kit (23,225, Thermo Fisher Scientific, Waltham, MA) according to the manufacturer’s protocol. Protein levels were analyzed using automated Western blotting with Wes™ (ProteinSimple, Santa Clara, CA) using the primary antibody of UCP1 (#14670; Cell Signaling) and phosphorylated cAMP response element-binding protein (CREB) (Ser133, #9198; Cell Signaling). Protein expression levels were normalized to β-actin (#8457; Cell Signaling) housekeeping protein expression. Western blot quantifications were done with Image J software.

### Statistical analysis

All statistical analyses were performed using GraphPad Prism 9 (GraphPad Software Inc., CA). Unpaired two-tailed Student's *t* test was used for comparisons, and *P* values less than 0.05 were regarded as statistically significant. All data are shown as mean ± SEM.

## Results

### S.tm infection induces a febrile response coinciding with elevated fat oxidation and decreased plasma TGs in APOE∗3-Leiden.CETP mice

**Fig. 1:**
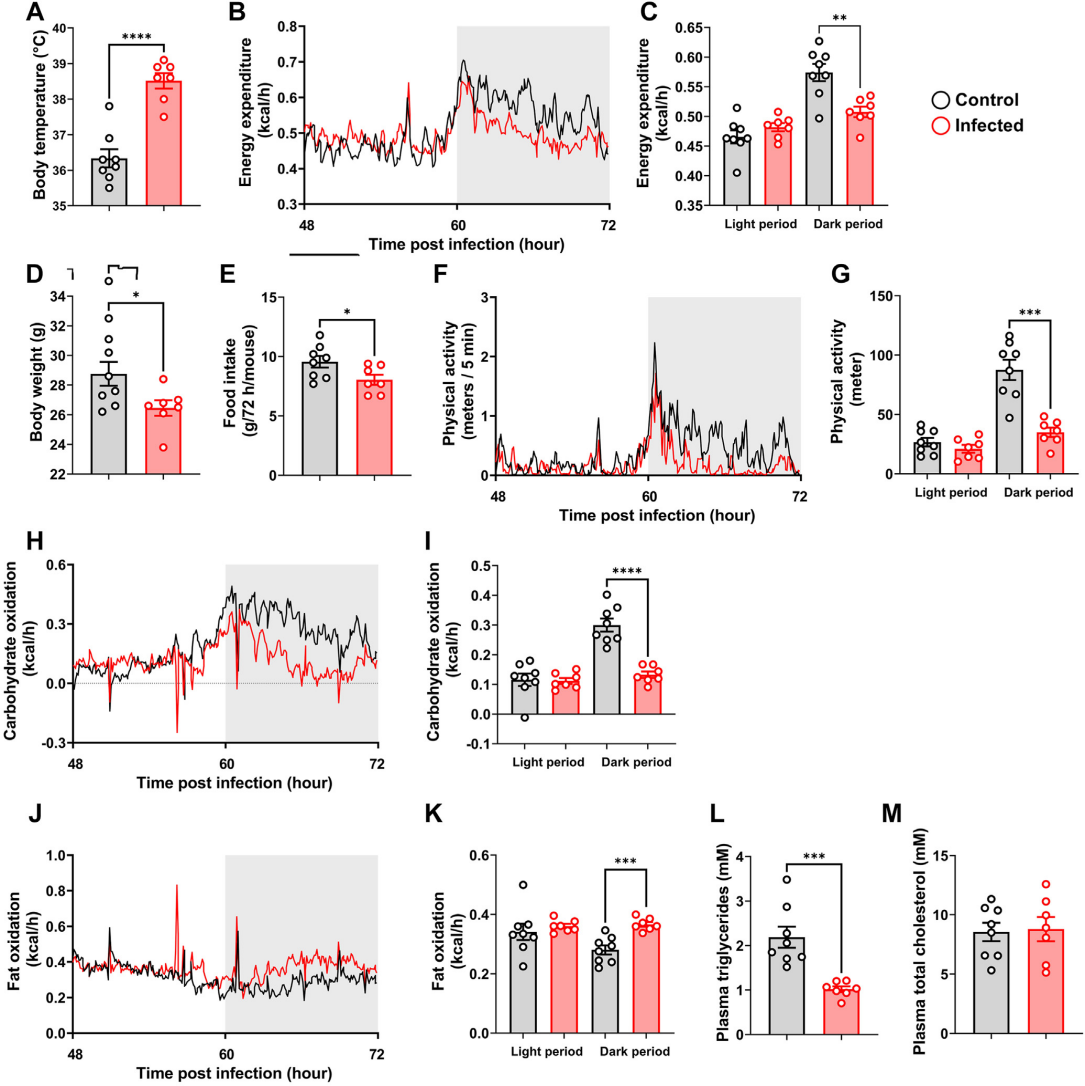
S.tm infection increases body temperature and induces fat oxidation in APOE∗3-Leiden.CETP mice. Male APOE∗3-Leiden.CETP mice were intraperitoneally injected with S.tm or vehicle as control. (A) Body temperature measured 72 h postinfection. (B, C) Energy expenditure 48–72 h postinfection. (D) Body weight 72 h post infection. (E) Total food intake during 72 h post infection. (F, G) Physical activity, (H, I) carbohydrate oxidation, and (J, K) fat oxidation as determined by indirect calorimetry 48–72 h postinfection. (L) Plasma triglycerides and (M) plasma total cholesterol levels 72 h postinfection. Data are presented as mean ± SEM (n = 7–8 mice/group). ∗*P* < 0.05, ∗∗*P* < 0.01, ∗∗∗*P* < 0.001, ∗∗∗∗*P* < 0.0001 according to an unpaired two-tailed Student's *t* test.

Seventy-two hours post infection of APOE∗3-Leiden.CETP mice with S.tm, mice exhibited a higher body temperature compared to noninfected control mice (38.5 ± 0.2°C vs. 36.3 ± 0.3°C; Fig. 1A). Whole-body energy expenditure, however, was largely reduced on the third day postinfection, in particular during the final 12-h observation period (Fig. 1B, C). This was attributed to both, a lower body weight (26.5 ± 0.5 g vs. 28.8 ± 0.8 g; Fig. 1D) and reduced food intake (8.0 ± 0.4 g vs. 9.6 ± 0.5 g; Fig. 1E), alongside with reduced physical activity during the final 12-h dark period (Fig. 1F, G). While carbohydrate oxidation was strongly decreased in the infected mice (Fig. 1H, I), fat oxidation was elevated (Fig. 1J, K). This shift in substrate oxidation was accompanied by a significant reduction of plasma TG levels in the infected mice (1.0 ± 0.1 vs. 2.2 ± 0.2 mmol/L; Fig. 1L), without concomitant alterations of plasma TC levels (Fig. 1M).

### S.tm infection promotes TG-derived FA uptake by BAT and WAT and attenuates cholesterol-enriched remnant uptake by the liver in APOE∗3-Leiden.CETP mice

**Fig. 2:**
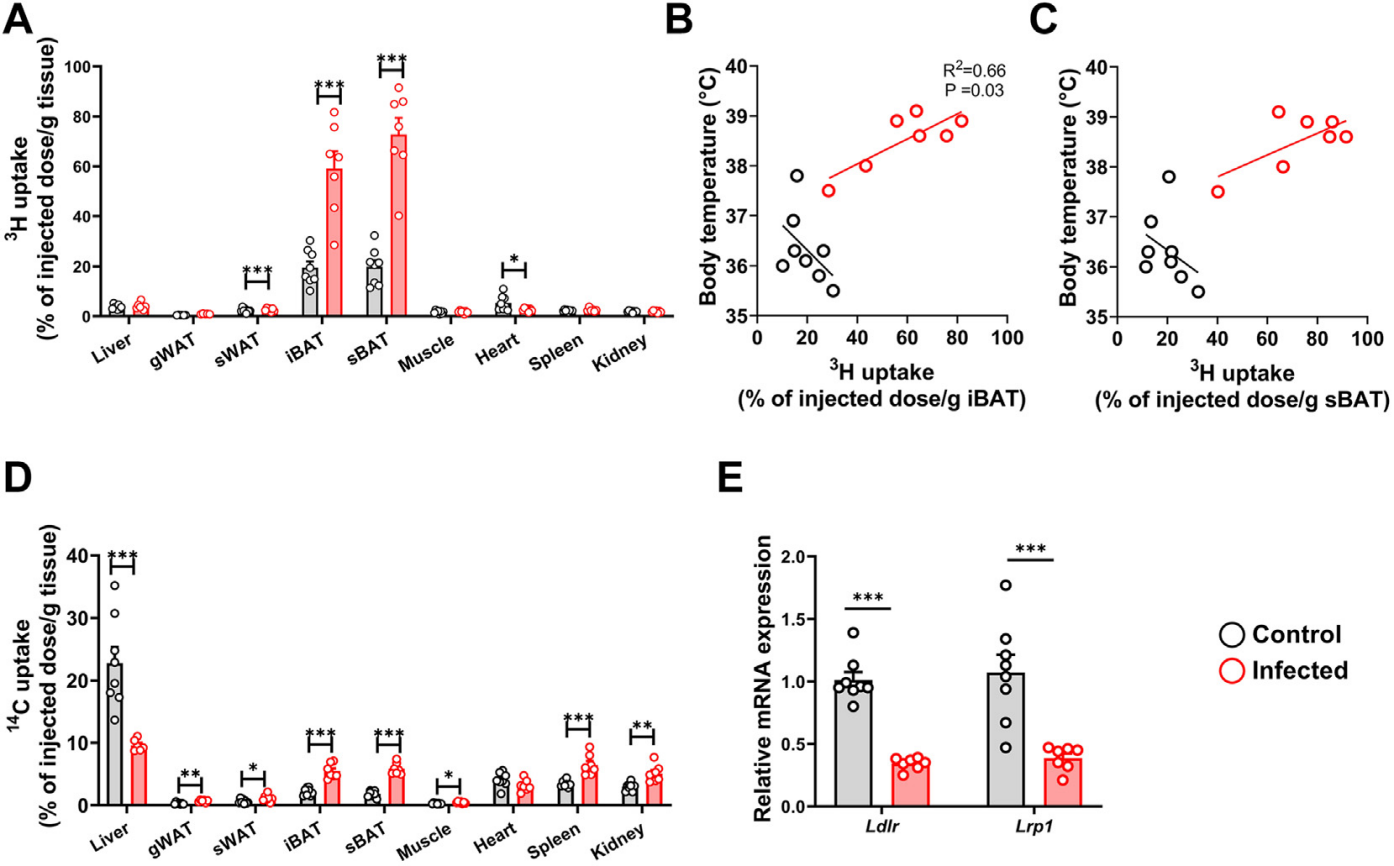
S.tm infection promotes triglyceride-derived fatty acid uptake by brown adipose tissue in APOE∗3-Leiden.CETP mice. Three days postinjection with S.tm or vehicle, mice were injected with triglyceride (TG)-rich lipoprotein (TRL)-like particles double-labeled with glycerol tri[^3^H]oleate and [^14^C]cholesteryl oleate. (A) ^3^H-activity in liver, gonadal white adipose tissue (gWAT), subcutaneous WAT (sWAT), interscapular brown adipose tissue (iBAT), subscapular BAT (sBAT), soleus (muscle), heart, spleen, and kidney. Core body temperature plotted against ^3^H-activity in (B) iBAT or (C) sBAT. (D) ^14^C-activity metabolic tissues. (E) Hepatic expression of *Ldlr* and *Lrp1*. Data are presented as means ± SEM (n = 7–8 mice/group). ∗*P* < 0.05, ∗∗*P* < 0.01, ∗∗∗*P* < 0.001, according to an unpaired two-tailed Student's *t* test.

As a substantial decline in plasma TG levels was observed following S.tm infection in APOE∗3-Leiden.CETP mice, we investigated TG-derived FA uptake by metabolic organs. Thereto, mice were intravenously injected with TRL-mimicking particles labeled with [^3^H]TO and [^14^C]CO. In noninfected control mice, TG-derived FA uptake (i.e., [^3^H]TO-derived activity) was higher in various BAT compartments as compared to other tissues and varied between 10% and 35% of the injected dose per gram tissue. FA uptake by BAT was markedly increased in the infected mice and accounted for 25%–95% of the injected dose per gram BAT (Fig. 2A). In addition, S.tm infection caused a modest but significant increase in TG-derived FA uptake by sWAT (2.7 ± 0.6% vs. 2.3 ± 0.8%; Fig. 2A), while uptake by the heart was slightly diminished (2.6 ± 0.6% vs. 5.3 ± 3.0%; Fig. 2A). Interestingly, TG-derived FA uptake by iBAT was found to be positively correlated with core body temperature (*R*^2^ = 0.66; Fig. 2B) in the infected mice but not in the control mice. A similar relationship, albeit not significant, was observed between FA uptake by sBAT and core body temperature of the infected mice (*R*^2^ = 0.46, *P* = 0.09; Fig. 2C). No correlation was observed for any of the other collected tissues (supplemental Fig. S1A–C).

Higher TG-derived FA uptake from TRLs by thermogenic adipose tissue is usually coupled to increased TRL remnant uptake by the liver (14). However, we observed largely reduced uptake of [^14^C]CO-derived activity in the livers of the infected mice (22.8 ± 7.2% vs. 47.6 ± 13.0%; Fig. 2D). This effect was partly explained by retention and/or higher uptake of particle remnants in BAT and WAT depots and by slightly elevated uptake of cholesterol-enriched remnants by the spleen and kidney (Fig. 2D). A small negative correlation between [^14^C]CO levels in sWAT and core body temperature of infected animals was observed (supplemental Fig. S1F). Yet, the reduced uptake of remnants by the liver seemed to be primarily explained by a significant decrease in hepatic expression of low density lipoprotein receptor (*Ldlr*) (−66%, Fig. 2E) and low density lipoprotein receptor-related protein 1 (*Lrp1*) (−62%, Fig. 2E) in the infected mice.

### S.tm infection might increase thermogenic capacity of adipose tissue via elevating sympathetic outflow in APOE∗3-Leiden.CETP mice

**Fig. 3:**
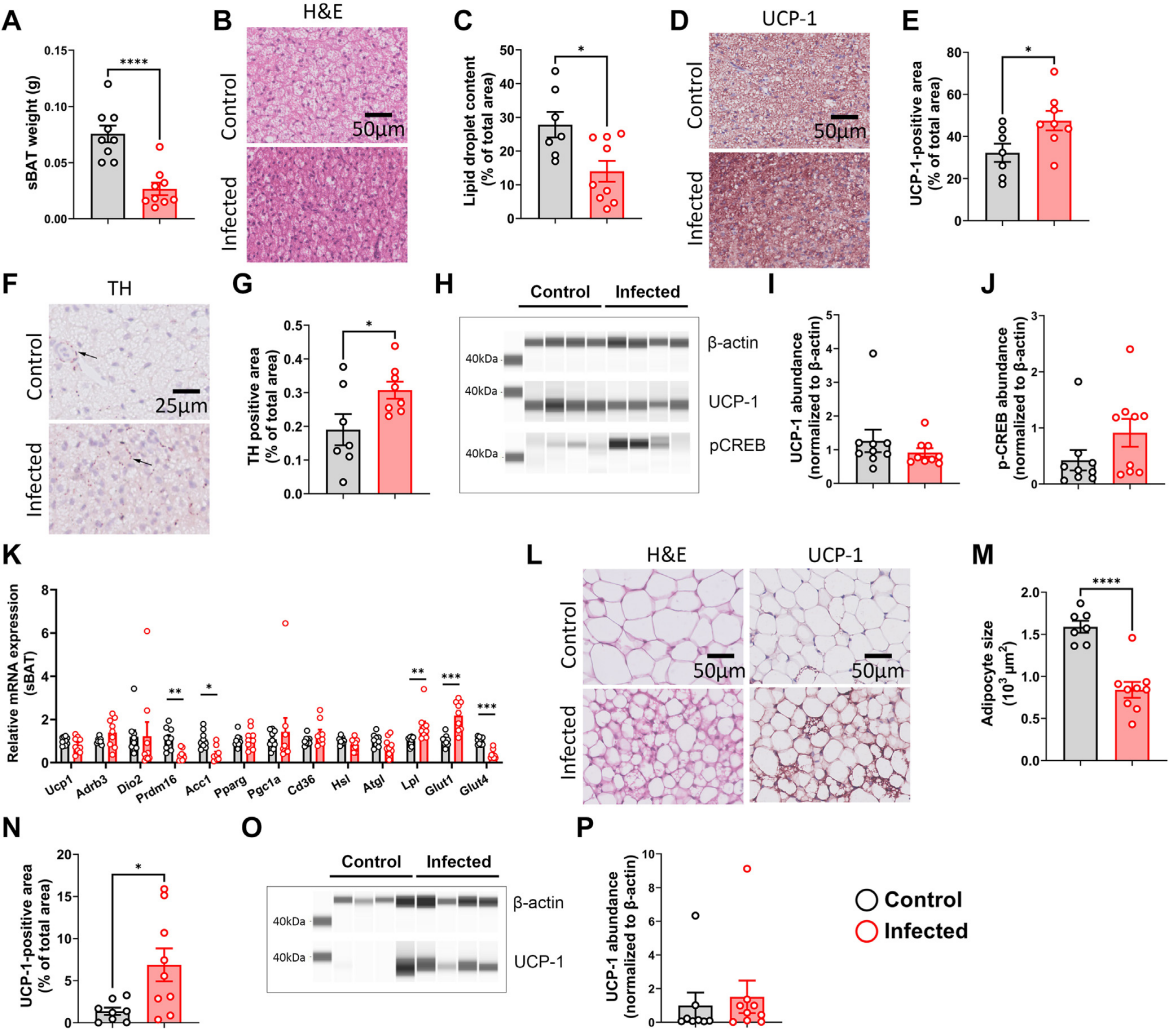
S.tm infection might increase thermogenic capacity of adipose tissue and sympathetic outflow toward BAT in APOE∗3-Leiden.CETP mice. (A) Weight of subscapular BAT (sBAT) 72 h postinfection. (B) Representative images of H&E staining in sBAT and (C) quantification of the lipid content within the tissue. (D) Representative images of immunostaining for uncoupling protein-1 (UCP-1) and (E) quantification thereof. (F) Immunostaining of tyrosine hydroxylase (TH) and (G) quantification thereof. (H) Protein abundance of UCP-1 and phosphorylated CREB in interscapular brown adipose tissue (iBAT) were measured by automated Western blot, and levels of (I) UCP-1 and (J) phosphorylated CREB were normalized to β-actin levels. (K) Gene expression of *Ucp1*, *Adrb3*, *Dio2*, *Prdm16*, *Acc1*, *Pparg*, *Pgc1a*, *Cd36*, *Hsl*, *Atgl*, *Lpl*, *Glut1*, and *Glut* 4 in sBAT. (L) Representative images of H&E and UCP-1 staining in subcutaneous WAT (sWAT), (M) quantification of adipocyte size and (N) quantification of UCP-1 content within the tissue. (O) UCP-1 protein abundance of sWAT and (P) quantification thereof. Values are presented as means ± SEM (n = 7–9 mice/group). ∗*P* < 0.05, ∗∗*P* < 0.01, ∗∗∗*P* < 0.001, ∗∗∗∗*P* < 0.0001 according to an unpaired two-tailed Student's *t* test. Acc, acetyl-CoA carboxylase; Lpl, lipoprotein lipase; Prdm16, PR domain containing 16.

Given the strongly increased uptake of TG-derived FAs by adipose tissues in infected mice compared to control mice, we next examined the morphology of these tissues. Three days postinfection, the weight of sBAT in infected mice was significantly lower when compared to that in control mice (0.03 ± 0.01 g vs. 0.08 ± 0.01 g; Fig. 3A). The tissue also appeared to be much denser (Fig. 3B), and the lipid droplet content was found to be strongly reduced in sBAT of the infected mice (14.0 ± 8.9% vs. 27.8 ± 10.0%; Fig. 3C). At the same time, a marked elevation in the abundance of UCP-1 content was observed (47.6 ± 13.0% vs. 32.3 ± 11.5%; Fig. 3D, E). Nevertheless, *Ucp1* gene expression measured by quantitative PCR (Fig. 3K) and UCP-1 protein levels measured by Western blotting (Fig. 3H, I) remained unchanged in S.tm infected mice. We continued by assessing the amount of TH as the rate-limiting enzyme in catecholamine production and as a marker for sympathetic activity. Infected mice showed elevated TH expression in sBAT when compared to noninfected mice (0.31 ± 0.07% vs. 0.19 ± 0.12%; Fig. 3F, G). S.tm infection also tended to increase phosphorylation of the transcription factor CREB (Fig. 3H, J), which is facilitated by protein kinase A downstream of the β-adrenergic receptor(s). On gene expression level (Fig. 3K), the relative expression of PR domain containing 16 and acetyl-CoA carboxylase was decreased, while expression of lipoprotein lipase was increased. Additionally, *glucose transporter 1* was upregulated, whereas *glucose transporter 4* was downregulated. Together, these data hint at a shift from FA synthesis to FA oxidation consistent with sympathetic innervation of the tissue.

Likewise, a reduction in adipocyte size was observed in sWAT (0.84 ± 0.30 vs. 1.59 ± 0.19 μm^2^; Fig. 3L, M) accompanied by the appearance of multilocular brown-like cells and expression of UCP-1 (6.9 ± 1.9% vs. 1.4 ± 0.4%, Fig. 3L, N). UCP1 protein as measured by Western blotting was found to be detectable in 6/9 of the infected mice versus 2/8 of the control mice (Fig. 3O, P).

### S.tm infection promotes thermogenic activity in adipose tissue of chow-fed wild-type C57BL/6J mice

**Fig. 4:**
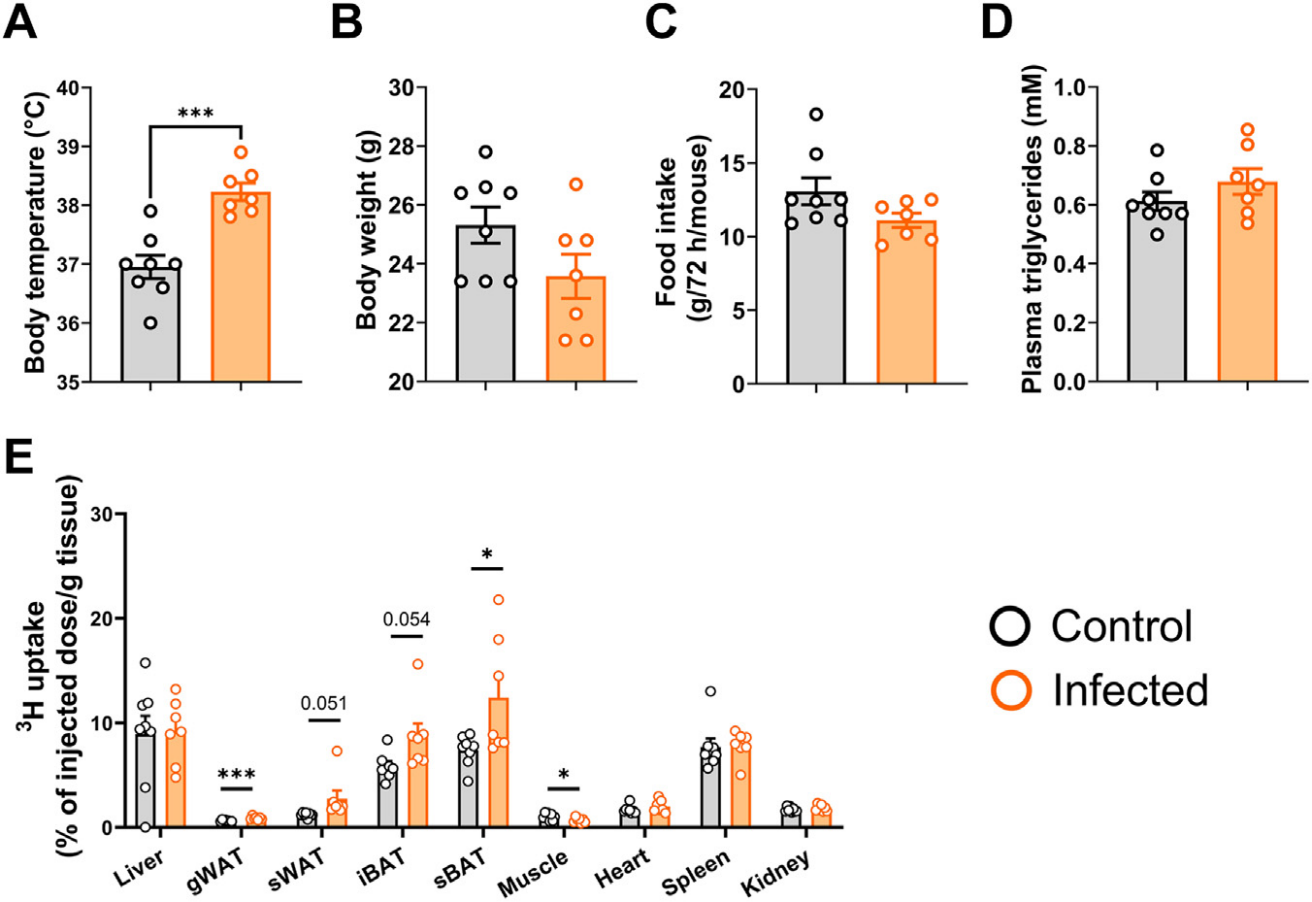
S.tm infection promotes triglyceride-derived fatty acid uptake by BAT in chow-fed, wild-type mice. Three days postinjection with S.tm or vehicle, wild-type mice (C57BL/6J background) were injected with triglyceride (TG)-rich lipoprotein (TRL)-like particles labeled with glycerol tri[^3^H]oleate, and (A) body temperature, (B) body weight, (C) total food intake, (D) plasma triglycerides, and (E) ^3^H-activity in liver, gonadal white adipose tissue (gWAT), subcutaneous WAT (sWAT), interscapular brown adipose tissue (iBAT), subscapular BAT (sBAT), soleus (muscle), heart, spleen, and kidney were determined. Data are presented as means ± SEM (n = 7–8 mice/group). ∗*P* < 0.05, ∗∗∗*P* < 0.001, according to an unpaired two-tailed Student's *t* test.

To confirm that our observations are not merely a feature of the APOE∗3-Leiden.CETP mice or a result of the Western-type diet feeding, we repeated the experiment in chow-fed, wild-type mice with the same C57BL/6J background. Seventy-two hours postinfection, S.tm-infected mice exhibited elevated body temperature compared to control mice (38.2 ± 0.1°C vs. 36.9 ± 0.2°C; Fig. 4A). Body weight, food intake, and plasma TG level, however, were not different between the two groups (Fig. 4B–D).

Similar to the results obtained from APOE∗3-Leiden.CETP mice, TG-derived FA uptake by BAT and WAT depots was increased upon infection of wild-type mice (Fig. 4E). In this specific experiment, we combined injection of [^3^H]TO-labeled TRL-like particles with 2-[^14^C]deoxyglucose and found slightly elevated ^14^C-activity in gonadal WAT, liver, and spleen of the infected mice (supplemental Fig. S2).

**Fig. 5:**
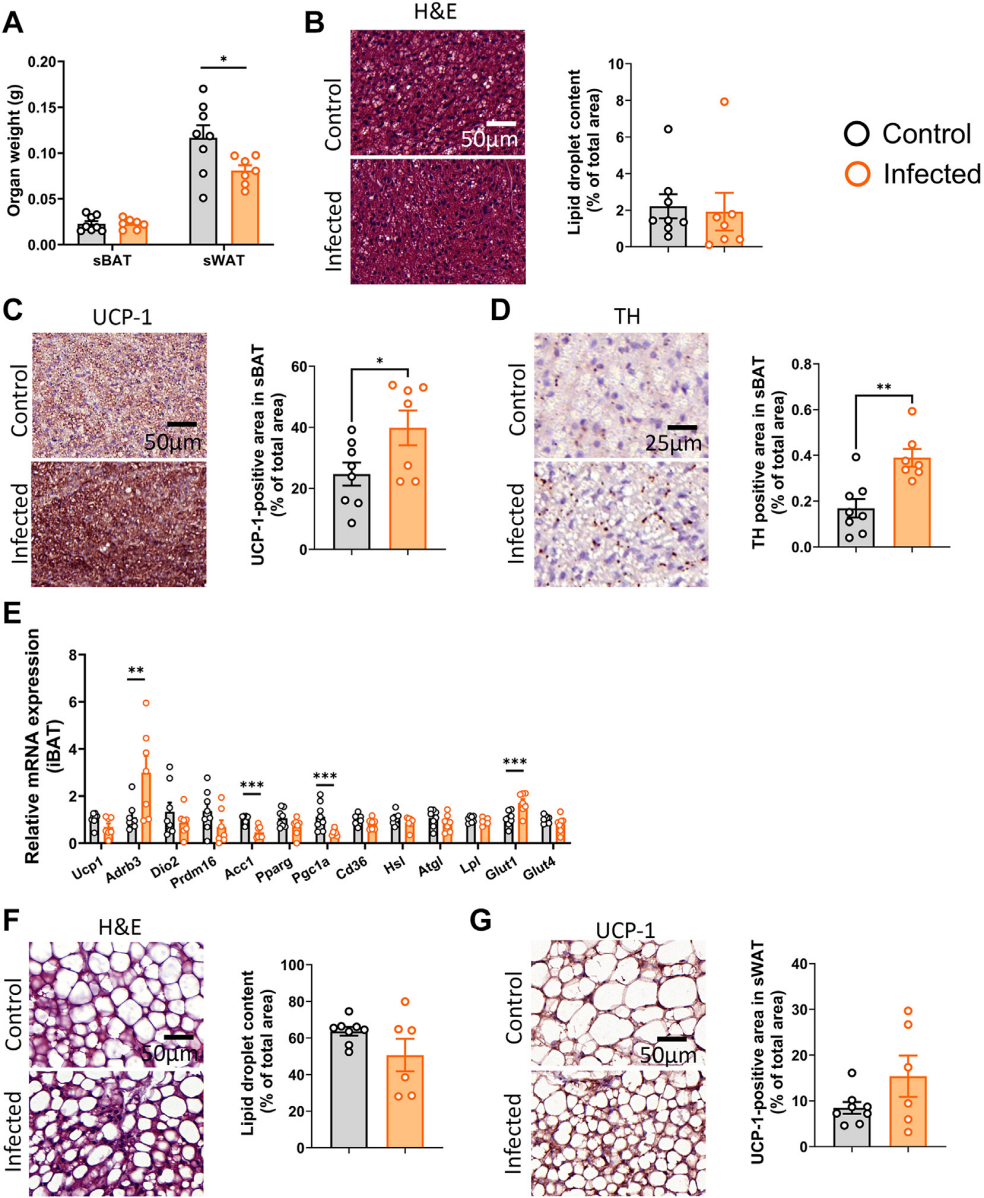
S.tm infection increases thermogenic capacity of BAT in chow-fed, wild-type mice. (A) Weight of subscapular BAT (sBAT) and subcutaneous WAT (sWAT) 72 h post S.tm infection. (B) Representative images of H&E staining in sBAT and quantification of the lipid content within the tissue. (C) Representative images of immunostaining in sBAT for uncoupling protein-1 (UCP-1) and quantification thereof. (D) Immunostaining of tyrosine hydroxylase (TH) in sBAT and quantification thereof. (E) Gene expression of *Ucp1*, *Adrb3*, *Dio2*, *Prdm16*, *Acc1*, *Pparg*, *Pgc1a*, *Cd36*, *Hsl*, *Atgl*, *Lpl*, *Glut1* and *Glut4* in interscapular brown adipose tissue (iBAT). (F) Representative images of H&E and quantification of lipid droplet content within subcutaneous WAT (sWAT) and quantification thereof. (G) UCP-1 staining in sWAT and quantification thereof. Values are presented as means ± SEM (n = 6–8 mice/group). ∗*P* < 0.05, ∗∗*P* < 0.01, ∗∗∗*P* < 0.001, according to an unpaired two-tailed Student's *t* test. Acc1, acetyl-CoA carboxylase; Prdm16, PR domain containing 16; Glut4, glucose transporter 4.

Despite elevated TG-derived FA uptake, sBAT weight was not altered in infected wild-type mice (Fig. 5A), suggesting that FA oxidation must be elevated. Lipid content of sBAT was also not different between the groups (Fig. 5B). Yet, S.tm infection resulted in increased UCP-1 density compared to controls (39.8 ± 5.7% vs. 24.6 ± 3.8%, Fig. 5C), and this effect was accompanied by a two-fold increase in TH content (0.40 ± 0.04% vs. 0.17 ± 0.04%, Fig. 5D). Consistent with the results of the experiment in APOE∗3-Leiden.CETP mice, we found suppressed *acetyl-CoA carboxylase* and upregulated *glucose transporter 1* gene expression in BAT of infected wild-type mice (Fig. 5E). Interesting enough, we additionally observed elevated expression of the *Adrb3* and downregulated expression of *Pgc1α* in this model.

While total body weight was not different between infected and noninfected mice, tissue weight of sWAT was lower in the infected animals (Fig. 5A), although we could not directly link this with lipid content (50.6 ± 8.9% vs. 63.6 ± 2.5%, *P* = 0.13, Fig. 5F) or UCP-1 content (15.4 ± 4.5% vs. 8.5 ± 1.3%, *P* = 0.12, Fig. 5G) in histological sections.

## Discussion

In this study, we explored the role of BAT thermogenesis in infection-triggered fever in APOE∗3-Leiden.CETP mice, a well-recognized model for human-like lipoprotein metabolism. We demonstrated that S.tm infection stimulates fat oxidation, accompanied by a pronounced reduction in circulating TG levels but not of cholesterol levels. We explained this by strongly elevated TG-derived FA uptake from TRLs by BAT, while the uptake of cholesterol-enriched remnants by the liver was attenuated, as a result of downregulation of *Ldlr* and *Lrp1* expression in hepatocytes. Histological examination further indicated increased expression of thermogenic markers in BAT and WAT and elevated sympathetic input in BAT. Repeating the experiment in wild-type mice (on the same C57BL/6 background) demonstrated that the effects of infection on thermogenic activity in adipose tissue were not merely a result of the dyslipidemic background. Altogether, we provide evidence for a role of thermogenic adipose tissue in fever.

Infection with S.tm induced a set of characteristic symptoms in APOE∗3-Leiden.CETP mice, including elevated body temperature, decreased body weight, and reduced physical activity and food intake, all of which are commonly associated with infections. These effects were accompanied by a reduction in whole-body energy expenditure, but interesting enough fat oxidation rates were higher in infected mice compared to control mice. Although the increase in fat oxidation is consistent with BAT activation (15), prolonged fasting also contributes to such metabolic changes, and we therefore have to be careful when interpreting these data. While we report a striking reduction in circulating TG levels as a result of increased TG-derived FA uptake by BAT and WAT in the infected mice, the acute-phase response to different types of infections or inflammatory stimuli is typically characterized by elevated TG levels explained by increased lipolysis in WAT and increased very-low density lipoprotein production by the liver (16). It is conceivable that in the current study, 72-h post S.tm infection is beyond the acute-phase response (17) and/or that the TG stores in WAT are already largely exhausted. In line with this notion, recent studies demonstrated a reduction in TG levels in induced mouse sepsis model employing cecal ligation (18), as well as in mice 15-weeks postinfection with *Yersinia pseudotuberculosis* (19). The fact that we observed elevated TG-derived FA uptake by adipose tissues, without a concurrent increase in tissue weight, strongly suggests that these tissues must be using these FAs as an energy source for heat production, thereby contributing to the observed increase in whole-body fat oxidation upon S.tm infection. This idea is further supported by the positive correlation between TG-derived FA uptake by BAT and core body temperature in infected mice. The lowered uptake of cholesterol-enriched lipoprotein remnants by the liver as a results of lowered *Ldlr* and *Lrp1* expression upon infection has been reported before (20) and probably intents to increase the availability of those lipids as sources of energy for other tissues or cells. This would be in line with our observation of increased uptake of lipids remnants by the spleen of infected mice.

Despite the positive correlation between metabolic activity in BAT and core body temperature, the question remains to what extent adipose tissue thermogenesis can contribute to body temperature during infection. Development of the fever involves the activation of heat production mechanisms (i.e., by increased shivering thermogenesis and activated nonshivering thermogenesis) and prevention of heat loss (i.e., by skin vasoconstriction, piloerection, and decreased sweating) (21, 22). Previous studies suggested no major role for BAT activation in fever induction following LPS or IL-1β injection (7, 8), although this may have been related to the specific models and/or stimuli used or the specific time points having been investigated. For example, Eskilsson *et al.* observed no elevation in the expression of *Ucp1* or *Pgc1a* mRNA, and no noticeable changes in BAT weight or TG levels following the administration of LPS (7), but they assessed UCP-1 expression only 4 hours after LPS injection and analyzed weight of BAT only 6 hours after the onset of endotoxemia. Similarly, Okamatsu-Ogura *et al.* did not detect any alterations in *Ucp1* expression, BAT weight, or TG levels at 1 hour post IL-1β injection (8). This is also consistent with the idea that fever induction in mice is primarily attributed to a reduction in heat dissipation rather than being due to acute initiation of thermogenesis (23). We anticipate that thermogenesis by BAT may become gradually more important during the course of infection. We should also realize that during adrenergic stimulation of brown adipocytes, free FAs generated from lipolysis cat activate UCP-1 and promote uncoupled respiration without a significant increase in its expression. In addition, we have to be cautious when interpreting results of experiments with UCP-1 knockout mice which did not show clear alterations in infection-induced fever responses (7, 8). Apart from the early single time point investigated in those studies, UCP-1 knockout mice can tolerate low environmental temperatures and are not obese due to compensation by UCP-2 and the existence of UCP-1 independent futile cycles (24).

In our study, S.tm-infected mice elicited an elevation in SNS outflow toward BAT, evidenced by a rise of TH expression, a trend for increased phosphorylated CREB downstream of adrenergic receptor signaling, and a gene expression signature consistent with sympathetic stimulation of this tissue. Elevated lactate levels as a marker for transition from simple infection to sepsis and switch from aerobic to anaerobic glycolysis, as well as inflammatory markers such as interleukin-6 have been reported to promote sympathetic outflow (25, 26). IL-1β and interleukin-6 bind cognate receptors on brain endothelial cells. This in turn elicits intracellular signaling, resulting in prostaglandin E2 synthesis (27) and stimulation of prostaglandin EP3 receptors in the median preoptic nucleus of the hypothalamus, ultimately leading to a cascade of fever responses including both, increased thermogenesis through activation of BAT and reduced passive heat loss through the skin by tail artery vasoconstriction (28).

In addition to activation of BAT, we also found increased TG-derived FA uptake by WAT accompanied by higher UCP-1 protein content in BAT of infected APOE∗3-Leiden.CETP mice, indicative of a phenomenon called browning of WAT. Together these findings support the idea that an escalating SNS outflow toward BAT and WAT is responsible for activation of thermogenic processes in the tissues following infection. Additionally, the browning phenomena and alteration in lipid metabolism indicate an overall activated SNS. Likewise, a prior study utilizing a cecal ligation polymicrobial sepsis model also described browning of adipose tissue in mice (29). However, obese mice have been reported to exhibit resistance to both, sepsis-induced and norepinephrine-induced WAT browning, while they experience reduced severity of illness (18). The resistance to infection-induced browning of adipose tissue in obesity can, at least in part, be attributed to a reduced sensitivity of white adipocytes to adrenergic stimuli which was also observed in obese patients (30).

Repeating the experiment in chow-fed wild-type mice resulted in a more or less similar stimulation of thermogenic activity in adipose tissue following S.tm infection. Yet, we did not observe changes in BAT weight or lipid content of the tissue. This likely relates to the fact that under normolipidemic conditions, lipid content is already at a very low level in these mice, but a limitation of the experimental design was that we could not discriminate between a genotype or diet effect. We did however observe a doubled TH content and significantly elevated UCP-1 density in BAT of the infected wild-type mice, indicative of adrenergic activation of the tissue.

In summary, our study demonstrated that bacterial sepsis induced by infection of S.tm leads to a rise in body temperature and fat oxidation, accompanied by a decrease in plasma TG levels. Furthermore, we observed a notable positive correlation between BAT activity and body temperature during the infection, suggesting that BAT thermogenesis might contribute to the fever response triggered by the infection. Additionally, SNS activity directed toward thermogenic adipose tissue were linked to increased BAT function and WAT browning. Based on our findings, we postulate that BAT, or adipose tissue thermogenesis in general, is involved in sustaining the elevated body temperature in the course of sepsis.

## Data availability

The data that support the findings of this study are available from the corresponding author upon reasonable request.

## Supplemental data

This article contains supplemental data.

## Conflict of interest

The authors declare that they have no conflicts of interest with the contents of this article.
