# Supplementary material for: Brown adipose tissue facilitates the fever response following infection with Salmonella enterica serovar Typhimurium in mice
Source: J Lipid Res. 2024 Aug 9;65(9):100617. doi: 10.1016/j.jlr.2024.100617 (PMC11407925; doi:10.1016/j.jlr.2024.100617)
Supplement: Supplemental data [file mmc1.docx]

**Supplementary material to**

**Brown adipose tissue facilitates the fever response following infection with *Salmonella enterica* serovar Typhimurium in mice**

**
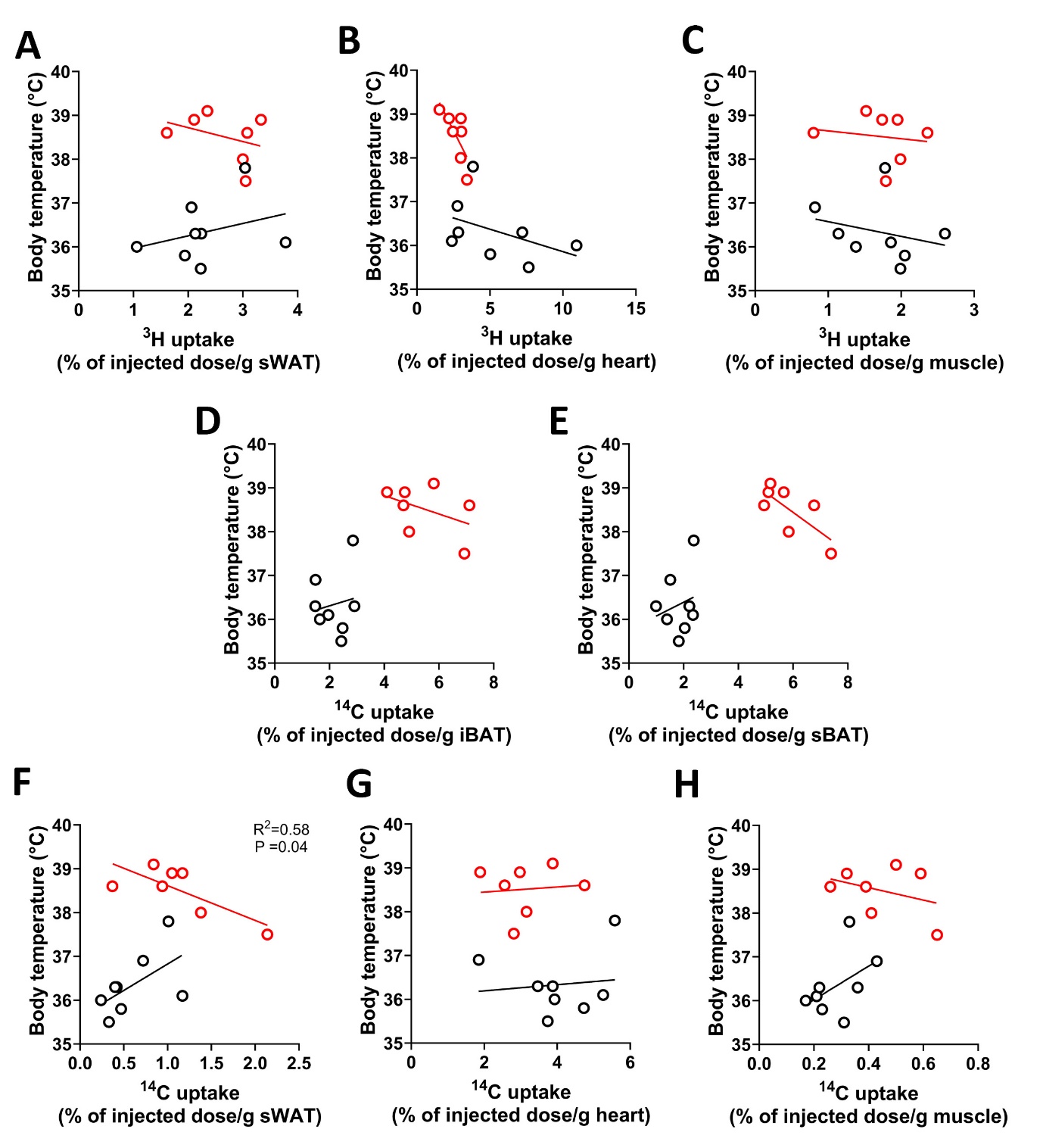
**

**Figure S1:** Male APOE*3-Leiden.CETP mice were intraperitoneally injected with *Salmonella enterica serovar* Typhimurium (S.tm) or vehicle as control. Three days post injection with S.tm or vehicle, mice were injected with triglyceride (TG)-rich lipoprotein (TRL)-like particles double-labeled with glycerol tri[^3^H]oleate and [^14^C]cholesteryl oleate. Core body temperature plotted against ^3^H-activity in (A) sWAT, (B) heart, and (C) muscle. Core body temperature plotted against ^14^C-activity in (D) iBAT, (E) sBAT, (F) sWAT, (G) heart and (H) muscle. n=7-8 per group.

**
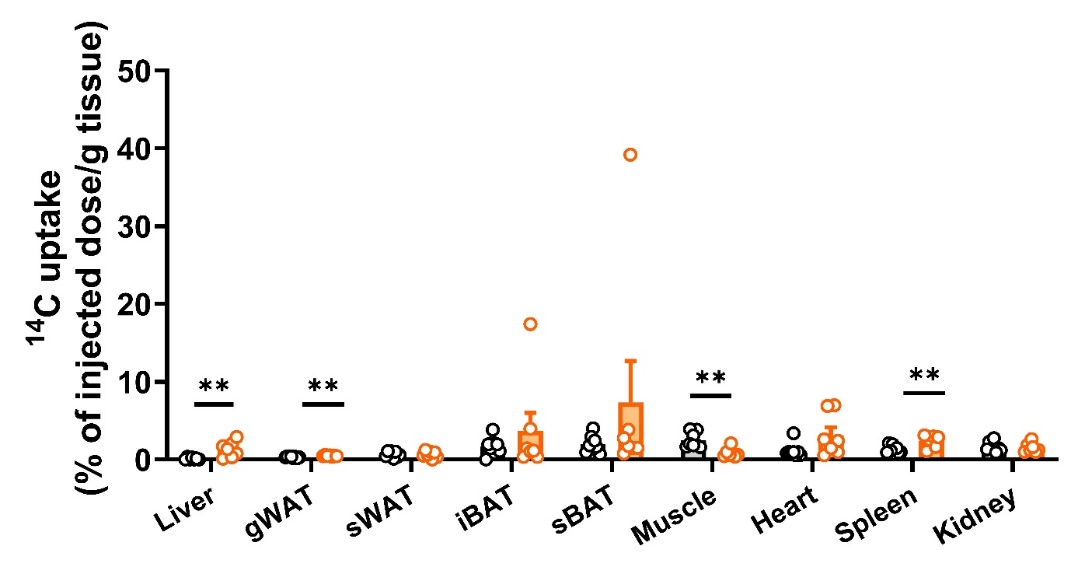
**

**Figure S2:** Male wild-type mice were intraperitoneally injected with S.tm or vehicle as control. Three days post injection with S.tm or vehicle, mice were injected with triglyceride (TG)-rich lipoprotein (TRL)-like particles labeled with glycerol tri[^3^H]oleate and 2-[1-^14^C]deoxy-D-glucose ([^14^C]DG). ^14^C-activity in liver, gonadal white adipose tissue (gWAT), subcutaneous WAT (sWAT), interscapular brown adipose tissue (iBAT), subscapular BAT (sBAT), soleus (muscle), heart, spleen, and kidney. Data are presented as means ± SEM (n = 7-8 mice/group). * p<0.05, ** p<0.01, according to an unpaired two-tailed Student's t-test.
